# Modified Ames test using a strain expressing human sulfotransferase 1C2 to assess the mutagenicity of methyleugenol

**DOI:** 10.1186/s41021-016-0028-x

**Published:** 2016-02-07

**Authors:** Hiroshi Honda, Kazuyuki Minegawa, Yurika Fujita, Noriko Yamaguchi, Yoshihiro Oguma, Hansruedi Glatt, Naohiro Nishiyama, Toshio Kasamatsu

**Affiliations:** R&D Safety Science Research, Kao Corporation, 2606 Akabane, Ichikai–Machi, Haga–Gun, Tochigi 321-3497 Japan; Tokyo Laboratory, BoZo Research Center Inc., 1-3-11, Hanegi, Setagaya-Ku, Tokyo 156-0042 Japan; Department of Nutritional Toxicology, German Institute of Human Nutrition (DIfE) Potsdam-Rehbruecke, Arthur-Scheunert-Allee 114-116, D-14558 Nuthetal, Germany

**Keywords:** Ames test, Methyleugenol, Alkenylbenzene, Sulfotransferase, S9

## Abstract

**Introduction:**

Several alkenylbenzenes, including methyleugenol (ME), are present in a wide range of botanicals and exhibit carcinogenic and mutagenic properties. Negative results are generally obtained for alkenylbenzenes in standard in vitro genotoxicity tests, including the Ames test. A lack of mutagenicity observed in such tests is thought to result from impaired metabolic activation of alkenylbenzenes via hydroxylation, with subsequent sulfoconjugation to its ultimate mutagenic or carcinogenic form. Although recent studies have reported the mutagenicity of hydroxylated ME metabolites in the Ames test using modified TA100 strains expressing human sulfotransferases (SULTs), to our knowledge, the detection of ME mutagenicity has not yet been reported.

**Findings:**

Using strain TA100-hSULT1C2, which expresses human SULT1C2, we optimized the protein content of S9 Mix and the pre-incubation time required to promote metabolic activation in the Ames test. This procedure enabled us to obtain a positive response with ME.

**Conclusions:**

We established Ames-test conditions enabling the detection of ME-induced mutagenicity, using a strain expressing human SULT1C2 in the presence of induced-rat S9 Mix. This simple approach will help assess the mutagenicity of other alkenylbenzenes and related chemicals.

## Findings

### Introduction

**Fig. 1:**
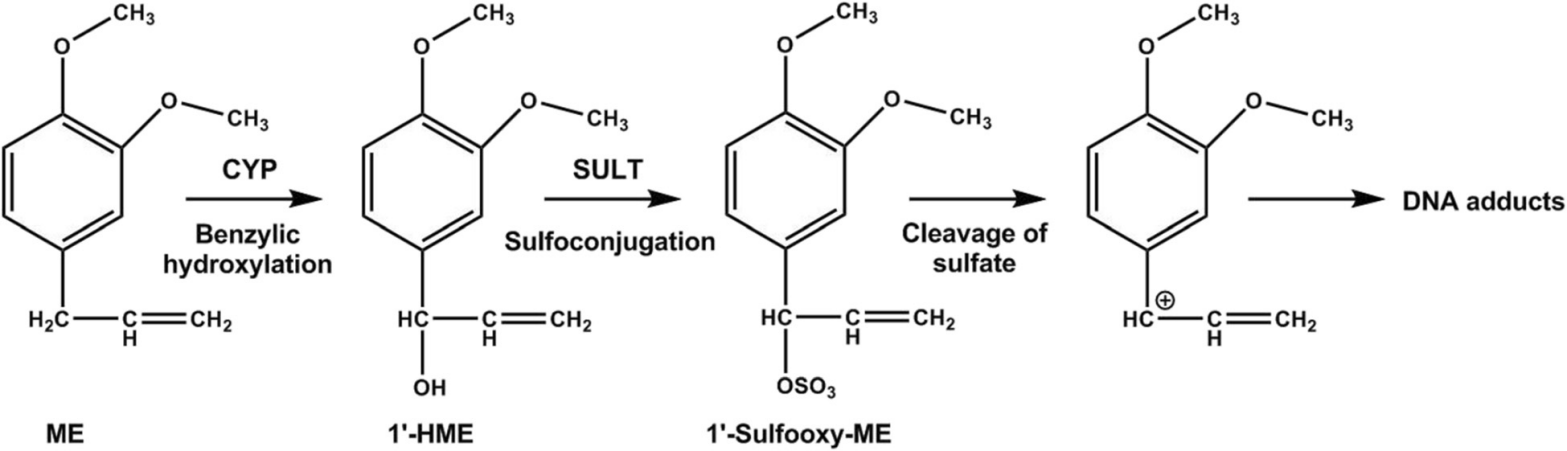
Metabolic-activation pathway of methyleugenol (ME) leading to the production of mutagenic metabolites. ME is metabolized by cytochrome P450 (CYP) to 1’-HME. Subsequent sulfoconjugation of 1’-HME by sulfotransferase (SULT) leads to the production of highly reactive electrophiles that can form DNA adducts

Alkenylbenzenes are present in a wide range of botanicals, including basil, nutmeg, and fennel, which are used in herbal teas, food flavorings, and food supplements. However, several alkenylbenzenes such as methyleugenol (ME), estragole, safrole, and β-asarone are known to exhibit mutagenicity [1] and hepatocarcinogenicity in rodents [2–4]. Findings from mechanistic studies indicate that alkenylbenzene-induced liver tumors result from the metabolism of these compounds to DNA-reactive intermediates. For example, ME is metabolized to 1’-hydroxymethyleugenol (1’-HME) by cytochrome P450 (CYP). Subsequent sulfoconjugation of 1’-HME by sulfotransferases (SULTs) leads to the production of highly reactive electrophiles that can form DNA adducts and thereby induce mutations (Fig. 1) [5, 6].

Human health-related concerns regarding the use of botanicals or botanical ingredients containing alkenylbenzenes have been raised; therefore, hazard assessments of alkenylbenzenes are of high priority [1]. However, the mutagenic properties of alkenylbenzenes are difficult to measure with standard in vitro genotoxicity assays, such as the bacterial reverse-mutation assay (Ames test) [7], due to a lack of specific enzyme activities in the exogenous metabolic system (rat S9 Mix) that is generally used in these assays.

Although several studies have shown that alkenylbenzenes are mutagenic [8–12], the experimental systems used require considerable effort or expertise when compared to standard genotoxicity assays (e.g., the Ames test). Therefore, a simple approach for utilizing standard genotoxicity assays, or a minor modification of such assays, is necessary.

Recently, Herrmann et al. demonstrated that hydroxylated metabolites of ME, including 1’-HME, are mutagenic in the Ames test when using *Salmonella typhimurium* TA100 strains expressing human SULTs [13]. However, detection of mutagenicity using the parent compound, ME, has not yet been reported in the Ames test. To assess the mutagenicity of alkenylbenzenes, we sought to establish Ames test conditions enabling the detection of mutagenicity of ME. As described above, ME is bioactivated in two steps, involving hydroxylation by CYP and sulfoconjugation by SULT. Employing the *S. typhimurium* strain TA100-hSULT1C2, which expresses human SULT1C2, we optimized the protein content of the S9 Mix and the pre-incubation time for co-incubating bacteria with test chemicals and the S9 Mix before the main incubation step, which promoted the generation of oxidized ME metabolites by CYP.

## Materials and methods

### Test strain

We used strain *S. typhimurium* TA100-SULT1C2, established by transforming the *S. typhimurium* TA100 strain with an expression vector, pKKneo-SULT, for the human *SULT1C2* gene [EMBL/DDBJ: AF186263] [14]. pKKneo-SULT was developed from cloning of human *SULT* cDNA into pKKneo (pKK233-2 [Stratagene] with the ampicillin-resistance marker replaced by a neomycin resistance marker) [14].

Several known mutagens exhibited strong mutagenic activity in TA100-hSULT1C2 but were inactive in the parental strain TA100 [13–16]. Among these mutagens, sulfoconjugation in furfuryl alcohol and 5-hydroxymethylfurfural has been demonstrated, and enzyme kinetic data have been established in cytosolic preparations from *hSULT1C2* expressed in *S. typhimurium* [17].

### Chemicals/reagents

JIS special grade dimethyl sulfoxide (negative-control solvent), 2-(2-furyl)-3-(5-nitrol-2-furyl)-acrylamide (AF-2; 99.6 % purity), and benzo[*a*]pyrene (B[*a*]P; 99.7 % purity) were used as positive-control reagents. These three reagents, as well as magnesium sulfate (>98 % purity), were obtained from Wako Pure Chemical Industries, Osaka Ltd. (Japan). Nutrient Broth No. 2 Culture Medium was purchased from OXOID Ltd. Hampshire (UK). Minimum-glucose medium was obtained from Kyokuto Pharmaceutical Industrial Co., Ltd. Tokyo (Japan). The protein contents were adjusted by changing the amount of the S9 fraction, prepared from 5,6-benzoflavone and phenobarbital-induced Sprague–Dawley rats, and water (total volume: 5 mL), and the mixtures were added to 10-mL vials of Cofactor-I (MgCl_2_: 16.3 mg; KCl: 24.6 mg; glucose-6-phosphate: 17.1 mg; NADPH: 34.3 mg; NADH: 30.3 mg; Na_2_HPO_4_: 119.6 mg; NaH_2_PO_4_/2H_2_O: 24.7 mg) (also see the *Optimization of the S9 Mix protein content* section).

### Ames test

We modified the pre-incubation method [18], as described previously [13]. Briefly, 30 mM magnesium sulfate was used instead of 0.1 mM sodium phosphate buffer (pH 7.4), and optimization of metabolic activation conditions was explored in terms of the protein content in the S9 Mix and pre-incubation times. Inorganic sulfate is required for the bacteria to synthesize 3′-phosphoadenosine-5′-phosphosulfate, the cofactor for sulfotransferases. Magnesium ions are activators of some enzymes involved in the synthesis of 3′-phosphoadenosine-5′-phosphosulfate.

To grow bacteria, 10 mL of Nutrient Broth No. 2 Culture Medium was placed into a sterilized, 40-mL capacity L-shape test tube, to which 10 μL of a bacterial suspension was added. Bacterial cultures were incubated at 37 °C with shaking at 100 rotations/min for 9 h before starting the experiment.

Ten microliters of test formulation, solvent, or positive-control reagent (AF-2 or B[*a*]P) solution was added to sterilized tubes. Next, 0.5 mL of 30 mM magnesium sulfate was added to the tubes for experiments not involving metabolic activation, or 0.25 mL of 60 mM magnesium sulfate and 0.25 mL of S9 Mix were added to promote metabolic activation. Subsequently, 0.1 mL of bacterial culture was added to each tube. Immediately after stirring, the tubes were pre-incubated for the indicated durations at 37 °C, with shaking at 80 rotations/min. Top agar (2.0 mL; 45 °C) was added and the resulting homogeneous mixtures were overlaid on agar plates containing Minimum-Glucose Medium. After the top agar solidified, the minimum-glucose agar plates were inverted in an incubator and cultured for 48 h at 37 °C. After incubation, the colonies were enumerated using an automatic colony counter (Colony Analyzer CA-11D systems, System Science Co., Ltd.). The bacteria were inspected under a stereomicroscope for the presence/absence of bacterial growth inhibition.

### Optimization of the S9 Mix protein content

Length of the pre-incubation time in this experiment was 1 h. For preliminary examinations, the S9 Mix was prepared with a total protein content of 4.0, 1.2 (common content for Ames tests), 0.3, or 0.1 mg protein/plate. Based on the results of the preliminary examinations, the main experiments were conducted using S9 Mix containing 2.4, 1.2, 0.6, or 0.3 mg of protein/plate.

### Optimization of the pre-incubation time

The concentration of the S9 fraction was set at 10 % (total protein content: 1.2 mg/plate), as normally used for Ames tests. The pre-incubation time was varied at 0 min, 20 min (ordinary time for the liquid pre-incubation version of the Ames tests), 1 h, and 2 h.

### Evaluation of results

We evaluated whether the number of revertant colonies in the presence of ME were increased by at least twice above that of the solvent control in a dose-dependent manner, which is a commonly used criterion for a positive response.

## Results and discussion

### Positive controls

To confirm that the experimental conditions other than the activity of hSULT1C2 work properly in the TA100-hSULT1C2 strain, we tested AF-2 without metabolic activation and B[*a*]P with metabolic activation under the same conditions as ME. As a result, both AF-2 and B[*a*]P yielded clear positive responses, indicating that the experimental conditions were valid (data not shown).

### Optimization of S9 Mix protein content

**Fig. 2:**
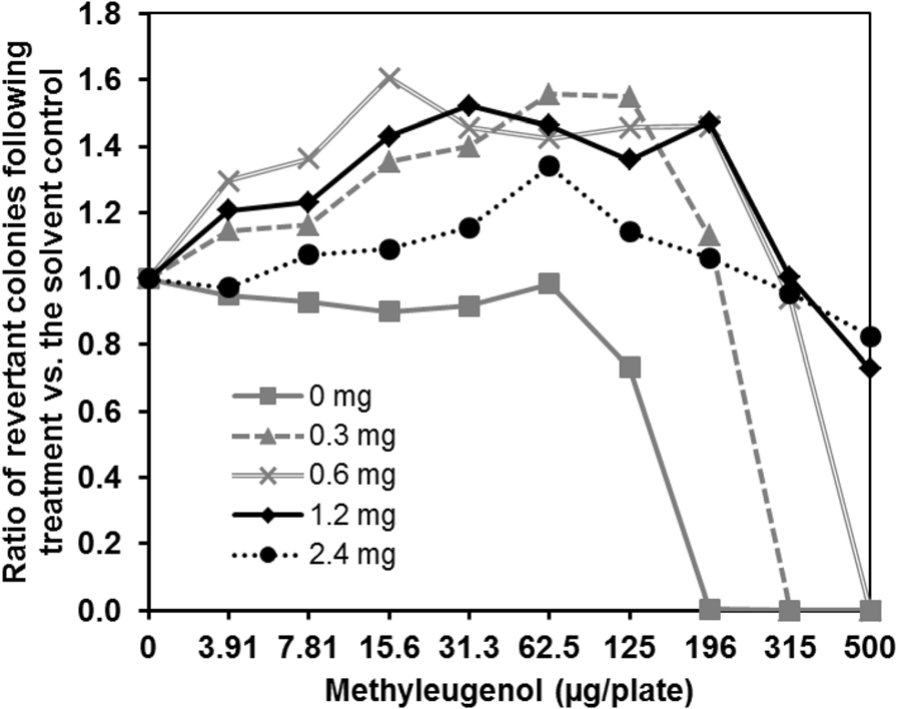
Optimizing the protein content in the S9 Mix. Based on the results of the preliminary investigation, the total protein contents in the S9 Mix were set at 0, 0.3, 0.6, 1.2, or 2.4 mg/plate. The pre-incubation time was set at 1 h. The plots indicate the ratio of the average number of revertant colonies observed (treated: solvent control) in two plates and the number of spontaneous revertants. The number of spontaneous colonies per plate ranged from 108 to 151, varying slightly in response to the different protein levels used

In preliminary experiments, slight increases in the number of revertant colonies were observed in ME treatment groups when the S9 Mix protein content was above 0.3 mg/plate (data not shown). Next, we prepared S9 Mix for subsequent experiments such that the protein content ranged from 0.3 to 2.4 mg/plate. The number of revertant colonies increased similarly regardless of protein content, but these increases were not sufficient to be judged as positive responses (2-fold increase over the solvent control; Fig. 2). Thus, our results suggested that the protein content offers diminishing returns above a threshold level.

### Optimization of the pre-incubation time

**Fig. 3:**
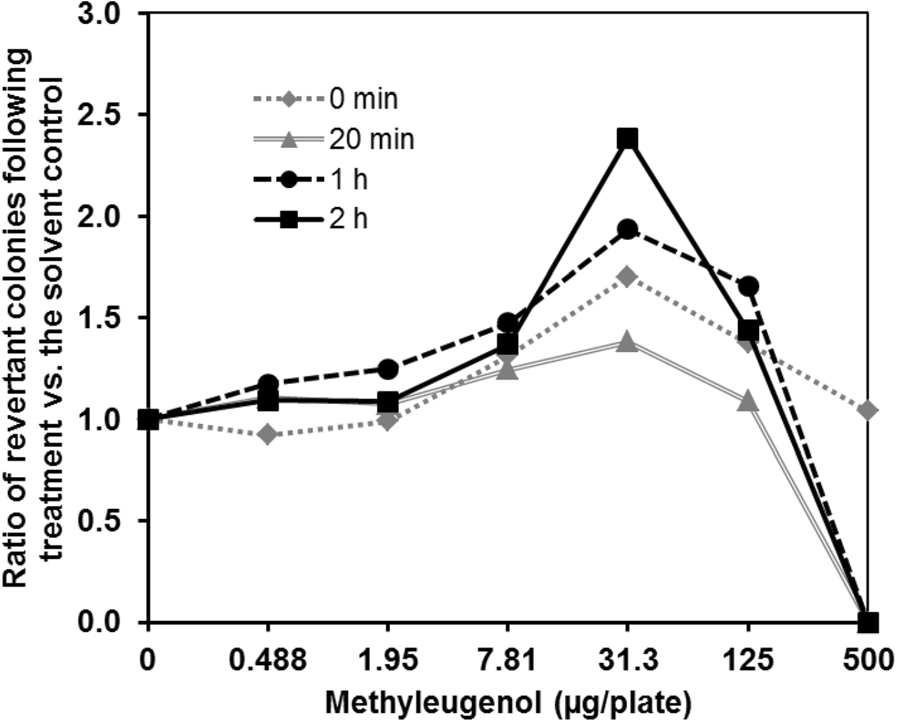
Optimization of the pre-incubation time. The pre-incubation time was set at 0 min, 20 min, 1 h, or 2 h. The protein content in the S9 Mix was set at 1.2 mg/plate. The plots shown indicate the ratio of the average number of revertant colonies observed (treated: solvent control) in two plates and the number of spontaneous revertants. The number of spontaneous colonies per plate ranged from 100 to 133, varying slightly in response to the different pre-incubation times used

**Fig. 4:**
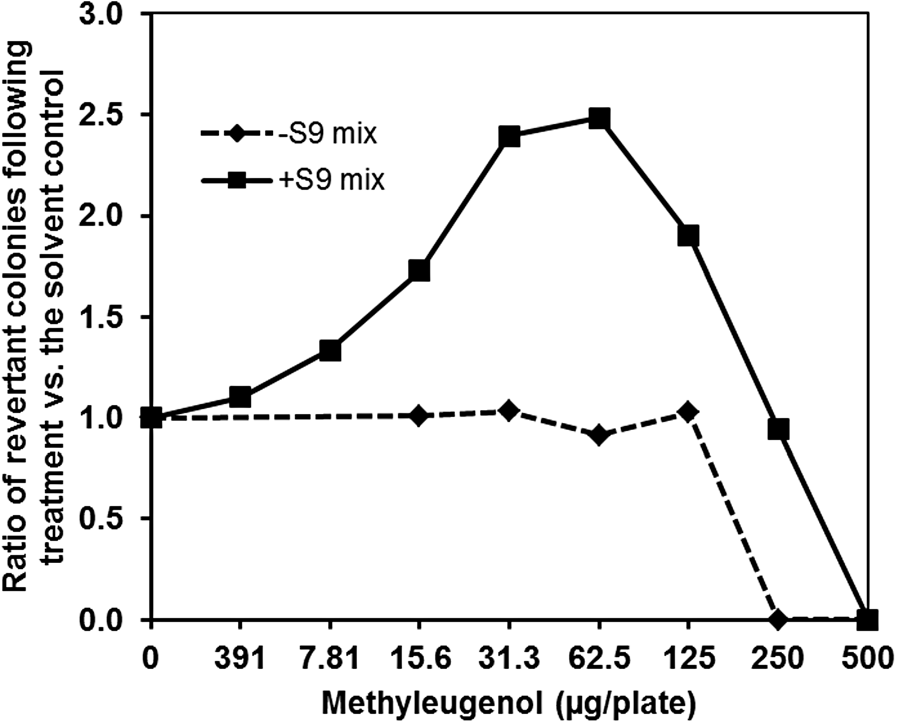
Results obtained under optimized conditions. The total protein content in the S9 Mix was set at 1.2 mg/plate (a common concentration for Ames tests), and the length of the pre-incubation period was 2 h for the main tests. The plots shown indicate the relative ratio of the average number of revertant colonies observed (treated: solvent control) in two plates and the number of spontaneous revertants. The number of spontaneous colonies per plate ranged from 96 to 128, varying slightly in the presence and absence of the S9 Mix in response to the different pre-incubation times used

Effects of varying pre-incubation time on the mutagenicity of ME are shown in Fig. 3. When the pre-incubation time was 2 h, the number of revertant colonies increased in a time-dependent manner to at least twice that of the negative-control group. Then, we conducted an Ames test for ME under the optimized condition (protein content: 1.2 mg/plate, pre-incubation time: 2 h), and a positive response was confirmed under the metabolic-activation condition (Fig. 4). Although the response was just above a 2-fold increase compared to the negative control group, the reproducibility of the positive response was confirmed (Figs. 3 and 4).

### Implication for evaluating the mutagenicity of alkenylbenzenes

To our knowledge, this is the first report describing the detection of the ME mutagenicity using the Ames test. Because 1’-HME mutagenicity has been detected previously using the TA100-SULT1C2 strain [13], prolonging the pre-incubation time may be effective in enhancing the production of mutagenic metabolites from ME by CYP and SULT. It should be noted that the approach used in the present study is primarily suited for hazard assessment, rather than risk assessment. Human SULT1C2, expressed in the TA100-hSULT1C2 cells used in this study, is a fetal enzyme that is rarely expressed in adult tissues [19]. Therefore, using the TA100 strain expressing human SULT1A1, which is abundantly expressed in many tissues in adult humans and shows considerable 1’-HME-sulfoconjugation activity [13], would be more relevant to human risk assessment. Indeed, genetic manipulation of the SULT1A status (knockout of the endogenous SULT1A gene or overexpression for the human SULT1A1/2 gene cluster) drastically affected the formation of hepatic DNA adducts in mice treated with ME or 1’-HME [20].

Furthermore, alkenylbenzene hydroxylation may vary with CYP expression levels or isoforms. Gardner et al. reported that the highest enzyme activities in human subjects were equivalent to that observed in untreated rats [21]. Cartus et al. found somewhat higher activities in human liver microsomes than in rat liver microsomes, in particular at low ME concentrations [22]. As we employed induced rat S9 Mix obtained from a rat administered 5,6-benzoflavone and phenobarbital, our system could potentially be optimized to promote ME hydroxylation and would be a very conservative approach in view of risk assessment. Safrole, another known mutagenic alkenylbenzene, is primarily 1’-hydroxylated by CYP2A6. CYP1A2 shows the highest activity in hydroxylating ME among the CYP enzymes [23]. Thus, careful attention is required when selecting a metabolic-activation system with an appropriate origin, taking into consideration structure-metabolism relationships. Nevertheless, the optimization of metabolic-activation conditions should be universally applicable.

## Conclusion

We established Ames test conditions that enable detection of a positive mutagenic response with ME. This simple approach will help assess the mutagenicity of alkenylbenzenes or other related chemicals.

## Abbreviations

1’-HME: 1’-hydroxymethyleugenol
AF-2: 2-(2-furyl)-3-(5-nitro-2-furyl)acrylamide
B[*a*]P: benzo[*a*]pyrene
CYP: cytochrome P450
ME: methyleugenol
SULT: sulfotransferase

## References

[1] van den Berg SJPL, Restani P, Boersma MG, Delmulle L, Rietjens IMCM (2011). Levels of genotoxic and carcinogenic ingredients in plant food supplements and associated risk assessment. Food Nutr Sci.

[2] National Toxicology Program (2000). NTP toxicology and carcinogenesis studies of methyleugenol (CAS NO. 93-15-2) in F344/N rats and B6C3F1 mice (gavage studies). Natl Toxicol Progr Tech Rep Ser.

[3] World Health Organization. IARC monographs on the evaluation of carcinogenic risk of chemicals to man volume 1. Natural products: safrole, isosafrole and dihydrosafrole. 1972. http://monographs.iarc.fr/ENG/Monographs/vol1-42/mono1.pdf. Accessed July 24, 2015.

[4] Miller EC, Swanson AB, Phillips DH, Fletcher TL, Liem A, Miller JA (1983). Structure-activity studies of the carcinogenicities in the mouse and rat of some naturally occurring and synthetic alkenylbenzene derivatives related to safrole and estragole. Cancer Res.

[5] IARC. IARC monographs on the evaluation of carcinogenic risks to humans volume 101. Some chemicals present in industrial and consumer products, food and drinking-water. Methyleugenol. 2012. http://monographs.iarc.fr/ENG/Monographs/vol101/mono101-013.pdf Accessed July 24, 2015. PMC4939416 24772663

[6] Rietjens IM, Boersma MG, van der Woude H, Jeurissen SM, Schutte ME, Alink GM (2005). Flavonoids and alkenylbenzenes: mechanisms of mutagenic action and carcinogenic risk. Mutat Res.

[7] Sekizawa J, Shibamoto T (1982). Genotoxicity of safrole-related chemicals in microbial test systems. Mutat Res.

[8] Randerath K, Haglund RE, Phillips DH, Reddy MV (1984). 32P-post-labelling analysis of DNA adducts formed in the livers of animals treated with safrole, estragole and other naturally-occurring alkenylbenzenes. I. Adult female CD-1 mice. Carcinogenesis.

[9] Burkey JL, Sauer JM, McQueen CA, Sipes IG (2000). Cytotoxicity and genotoxicity of methyleugenol and related congeners - a mechanism of activation for methyleugenol. Mutat Res.

[10] Zhou GD, Moorthy B, Bi J, Donnelly KC, Randerath K (2007). DNA adducts from alkoxyallylbenzene herb and spice constituents in cultured human (HepG2) cells. Environ Mol Mutagen.

[11] Jin M, Kijima A, Suzuki Y, Hibi D, Inoue T, Ishii Y (2011). Comprehensive toxicity study of safrole using a medium-term animal model with gpt delta rats. Toxicology.

[12] Oda Y, Zhang Y, Buchinger S, Reifferscheid G, Yang M (2012). Roles of human sulfotransferases in genotoxicity of carcinogens using genetically engineered umu test strains. Environ Mol Mutagen.

[13] Herrmann K, Engst W, Appel KE, Monien BH, Glatt H (2012). Identification of human and murine sulfotransferases able to activate hydroxylated metabolites of methyleugenol to mutagens in *Salmonella typhimurium* and detection of associated DNA adducts using UPLC-MS/MS methods. Mutagenesis.

[14] Meinl W, Pabel U, Osterloh-Quiroz M, Hengstler JG, Glatt HR (2006). Human sulphotransferases are involved in the activation of aristolochic acids and are expressed in renal target tissue. Int J Cancer.

[15] Glatt H, Schneider H, Murkovic M, Monien BH, Meinl W (2012). Hydroxymethyl-substituted furans: mutagenicity in *Salmonella typhimurium* strains engineered for expression of various human and rodent sulphotransferases. Mutagenesis.

[16] Meinl W, Tsoi C, Swedmark S, Tibbs ZE, Falany CN, Glatt H (2013). Highly selective bioactivation of 1- and 2-hydroxy-3-methylcholanthrene to mutagens by individual human and other mammalian sulphotransferases expressed in *Salmonella typhimurium*. Mutagenesis.

[17] Sachse B, Meinl W, Sommer Y, Glatt H, Seidel A, Monien BH. Bioactivation of food genotoxicants 5-hydroxymethylfurfural and furfuryl alcohol by sulfotransferases from human, mouse and rat: a comparative study. Arch Toxicol. 2014; Epub ahead of print 5 Nov 2014. 10.1007/s00204-014-1392-6 PMC4710668 25370010

[18] Matsushima T, Sugimura T, Nagao M, Yahagi T, Shirai A, Sawamura M, Norpoth KH, Garner RC (1980). Factors modulating mutagenicity in microbial tests. Short-term test systems for detecting carcinogens.

[19] Sakakibara Y, Yanagisawa K, Katafuchi J, Ringer DP, Takami Y, Nakayama T (1998). Molecular cloning, expression, and characterization of novel human SULT1C sulfotransferases that catalyze the sulfonation of N-hydroxy-2-acetylaminofluorene. J Biol Chem.

[20] Herrmann K, Engst W, Meinl W, Florian S, Cartus AT, Schrenk D (2014). Formation of hepatic DNA adducts by methyleugenol in mouse models: drastic decrease by Sult1a1 knockout and strong increase by transgenic human SULT1A1/2. Carcinogenesis.

[21] Gardner I, Wakazono H, Bergin P, de Waziers I, Beaune P, Kenna JG (1997). Cytochrome P450 mediated bioactivation of methyleugenol to 1’-hydroxymethyleugenol in Fischer 344 rat and human liver microsomes. Carcinogenesis.

[22] Cartus AT, Herrmann K, Weishaupt LW, Merz KH, Engst W, Glatt HR (2012). Metabolism of methyleugenol in liver microsomes and primary hepatocytes: pattern of metabolites, cytotoxicity, and DNA-adduct formation. Toxicol Sci.

[23] Jeurissen SM, Punt A, Boersma MG, Bogaards JJ, Fiamegos YC, Schilter B (2007). Human cytochrome P450 enzyme specificity for the bioactivation of estragole and related alkenylbenzenes. Chem Res Toxicol.

